# EEG-based BCI Dataset of Semantic Concepts for Imagination and Perception Tasks

**DOI:** 10.1038/s41597-023-02287-9

**Published:** 2023-06-15

**Authors:** Holly Wilson, Mohammad Golbabaee, Michael J. Proulx, Stephen Charles, Eamonn O’Neill

**Affiliations:** 1 Department of Computer Science, University of Bath, Bath, BA2 7AY UK; 2 Department of Engineering Mathematics, University of Bristol, Bristol, BS8 1TW UK; 3 Department of Psychology, University of Bath, Bath, BA2 7AY UK

**Keywords:** Neural decoding, Databases

## Abstract

Electroencephalography (EEG) is a widely-used neuroimaging technique in Brain Computer Interfaces (BCIs) due to its non-invasive nature, accessibility and high temporal resolution. A range of input representations has been explored for BCIs. The same semantic meaning can be conveyed in different representations, such as visual (orthographic and pictorial) and auditory (spoken words). These stimuli representations can be either imagined or perceived by the BCI user. In particular, there is a scarcity of existing open source EEG datasets for imagined visual content, and to our knowledge there are no open source EEG datasets for semantics captured through multiple sensory modalities for both perceived and imagined content. Here we present an open source multisensory imagination and perception dataset, with twelve participants, acquired with a 124 EEG channel system. The aim is for the dataset to be open for purposes such as BCI related decoding and for better understanding the neural mechanisms behind perception, imagination and across the sensory modalities when the semantic category is held constant.

## Background & Summary

Brain computer interfacing and cognitive neuroscience are fields which rely on high quality brain activity based datasets. Surface electroencephalography (EEG) is a popular choice of neuroimaging technique for BCIs due to its accessibility in terms of cost and mobility, its high temporal resolution and non-invasiveness. Although EEG datasets can be time consuming and expensive to obtain, they are extremely valuable. A single open source dataset can form the basis of many varied research projects, and thus can more rapidly advance scientific progress. For example, EEG datasets for inner speech commands^1^ and for object recognition^2^ were recently created and shared to address a lack of publicly available datasets in these areas. These datasets enable the development of sophisticated techniques for analysis and decoding, which can be used to investigate neural representation mechanisms and improve decoding performance for EEG based BCIs.

Different paradigms have been used for EEG based BCIs such as Event Related Potential (ERP) BCIs for decoding inner speech^1,3^, Steady-State Visual Evoked Potentials (SSVEPs)^4^ and motor imagery^5^, and oscillatory activity driven BCIs for tasks such as drowsiness detection^6^. Recently, there has been growing interest in decoding alternative information forms such as auditory and visual, perception and imagination^7^, and semantic information^8^. However, the lack of open source EEG datasets for decoding imagined and perceived semantic level information is hindering progress towards this research goal.

Visual decoding involves decoding simple low level visual components such as colour and shape, or complex naturalistic images of objects, scenes and faces. In contrast, semantic decoding extracts conceptual information such as object types or classes. For example, was the object in an image shown to an observer a flower or a guitar? The low level visual and auditory sensory details of the semantic concept, such as whether the flower is yellow or purple, are ignored with a focus on the high level meaning of ‘flower’. The advantage of decoding semantic information, as opposed to sensory based information such as visual details, is that semantic representation is partially invariant across modalities^9–13^. Invariance to low level sensory detail can be considered a desirable quality in BCI systems in which within class generalisabilty is a key goal. This can help increase robustness to real world data heterogeneity.

Growing evidence of neural overlap between perception and imagination^14,15^ may also facilitate generalisability. This task invariance has enabled cross-decoding between perception and imagination^16,17^. Efforts are being made to determine the spatiotemporal extent of these shared neural representations^18–20^, which may be most invariant in brain regions and time points associated with latent representations; i.e. closer to semantic level information. For example, the differences between imagery and vision appear to be most pronounced in the early visual cortex, with greater overlap occurring higher up in the visual hierarchy^21^ and at time points linked to high level perceptual processing^14^.

To drive decoding of a class based on semantic information, stimuli must vary in their low level sensory details. This approach was employed in a recent open source dataset that captured EEG measurements for object recognition using a rapid serial visual presentation paradigm^2^. The dataset includes 22,248 images related to 1,854 concepts. While there are impressive semantic decoding results emerging using fMRI^22–25^ and EEG^26–28^ which demonstrate feasibility, the field lacks an open source EEG dataset for researchers to investigate semantic representation across several sensory modalities, as well as both perception and imagination.

In this paper, we introduce a novel dataset, as well as the code for pre-processing and analysis, designed for investigating and decoding semantic representation of imagined and perceived visual and auditory information. We also present an initial analysis to demonstrate this dataset’s utility. To capture semantic representation, we drive high variance within each class (or rather semantic category). Specifically, we use three semantic concepts–penguin, guitar and flower–that participants perceived and subsequently imagined in auditory, visual orthographic, and visual pictorial forms. Furthermore, we provide a metric for the vividness of imagination metric for each participant for both the visual and auditory modalities. Individual differences in imagination capacity are shown to impact neural correlates^29,30^ and therefore may affect the decodability of, or the decoding strategy used for, each individual.

Some proposed uses of this dataset for both BCI and cognitive neuroscience oriented research questions include:

1. Decoding between sensory modalities such as auditory, visual orthographic and visual pictorial.
2. Decoding task type, specifically between perception and imagination.
3. Decoding the semantic category regardless of the sensory modality presentation or task.

## Methods

### Participants

Ethics approval was obtained from the Psychology Research Ethics Committee at the University of Bath (Ethics code: 19–302). Participants gave informed consent to take part in this study and for their data to be shared. Eight participants were recruited for a data collection pilot to ensure the quality of the dataset. This allowed us to identify and address any syncing issues with the Lab Streaming Layer network, as well as unexpected environmental noise at around 27 Hz in two of the sessions. The final version of the experiment was completed by twelve participants, most of whom were students at the University of Bath. Initially, selection criteria included normal or corrected vision and hearing, and excluded individuals with epilepsy. However, we later expanded the criteria to include individuals with visual and hearing impairments, to enable our dataset to support a wider range of research questions. One participant with visual and hearing impairment was included in the final sample. Participants were reimbursed £20 for their time in exchange for participating in an approximately two hour session.

**Table 1:** The Vividness of Visual Imagery Questionnaire (VVIQ) and Bucknell Auditory Imagery Scale (BAIS) scores for each participant, session and the average (avg) and standard deviation (std).

Metric	3_3	8_3	10_1	11_1	12_1	12_2	13_1	14_1	14_2	15_1	15_2	16_1	17_1	18_2	19_1	Avg	Std
VVIQ	4.25	3.1	4.9	3.3	3.2	3.2	—	4.2	4.2	4.1	4.1	—	3.5	3.6	3.1	3.75	0.55
BAIS	4.8	4.2	6.33	4	3.8	3.8	—	5	5	5.7	5.7	—	4	5.6	3.8	4.76	0.85

For two participants, sub-13 and sub-16, the vividness questionnaires were not completed.

### Experimental procedure

**Fig. 1:**
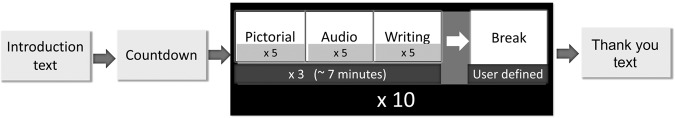
This figure shows an example of a pictorial trial. After a cue indicating whether the upcoming task is pictorial, orthographic or audio, five trials occur with a different stimulus used in each. Before the break, one block of each type of modality is cycled through, which takes around seven minutes. The duration of each break is chosen by the participant.

Participants were offered the opportunity to participate in a second data gathering session, in order to increase the number of trials for each participant. Of the twelve participants, nine completed one session and three returned for a second session. The experiment was conducted in a soundproof and lightproof room. It was not electrically shielded but all mains outlets other than the acquisition laptop charge point were turned off. The EEG setup, including cap fitting and gel application to the electrodes, took approximately 40 to 60 minutes. During the first session, participants completed two questionnaires while the gel was being applied: the vividness subscale of the Bucknell Auditory Imagery Scale (BAIS-V)^31^ and the Vividness of Visual Imagery Questionnaire (VVIQ)^32^. Subsequent to this, participants performed a practice version of the experimental tasks with a chance to ask questions around any uncertainties. After the setup was complete, the light was turned off, the experimenters left the testing room and went into an adjacent room, and the participant began the study when ready by pressing the computer keyboard’s space bar. For a schematic of the main task flow, see Fig. 1. The experiment was designed using Psychopy Version 3^33^, and presented on a 1920 × 1080 resolution screen. The Psychopy files are made available as described in the Usage Notes section. The ANT Neuro acquisition software ‘eego’ was used to record the EEG data. A Lab Streaming Layer (LSL) network sent the triggers from the presentation PC to the acquisition software to time-stamp the stimuli and task relevant information. There were ten blocks in total, though the majority of participants did not complete all the blocks due to fatigue or reporting reduced concentration. See Table 4 for the amount of trials completed for each condition for each participant. The participants were encouraged to take breaks between each block and call the experimenter if they required water or had any concerns.

### Questionnaire

The VVIQ and BAIS-V are self-report measures of mental imagery ability. The BAIS-V is a subscale of BAIS which captures the subjective clarity or vividness of an imagined sound, such as a trumpet playing happy birthday, on a scale of 1–7. A score of 7 is as vivid as the actual sound, whereas 1 indicates there was no sound at all. The VVIQ measures the subjective vividness of an imagined scenario such as a sunset, on a scale of 1–5, with 5 being the most vivid and 1 meaning no image at all. For VVIQ and BAIS-V results, see Table 1. The mean VVIQ score was 3.75 (std = 0.55) and average BAIS-V was 4.76 (std = 0.85). VVIQ and BAIS scores are significantly correlated as calculated using Spearmans Rank with r = 0.79 and p = 0.007.

### Data acquisition

A 128 channel *ANT Neuro eego Mylab* measuring system (ANT Neuro B.V., Hengelo, Netherlands) was used, with 124 EEG electrodes. The gel-based waveguard cap has active shielding which protects the signal from 50/60 Hz environmental noise. The sampling rate was 1024 Hz, with a 24-bit resolution. The montage, with pre-fixed electrode positions, is laid out according to the five percent electrode system^34^, which is an extension from the standard 10/20 layout for higher resolution EEG systems. The EEG cap size was selected based on the participant’s head circumference in cm. Large is 56–61 cm, medium is 51–56 cm and small is 47–51 cm. Once the cap was fitted to the participant’s head, OneStep Cleargel conductive gel was applied to the electrodes with CPz as reference, and the ground fixed to the left mastoid with Ten20 paste. Impedance of below 50 was sought, but due to variables such as hair thickness and other factors, there were often up to ten electrodes that had higher impedance. After the experiment was finished, the recording was stopped and the EEG data were stored as*.cnt* files, and the events as*.evt* files in ANT Neuro native format.

### The paradigms

**Fig. 2:**
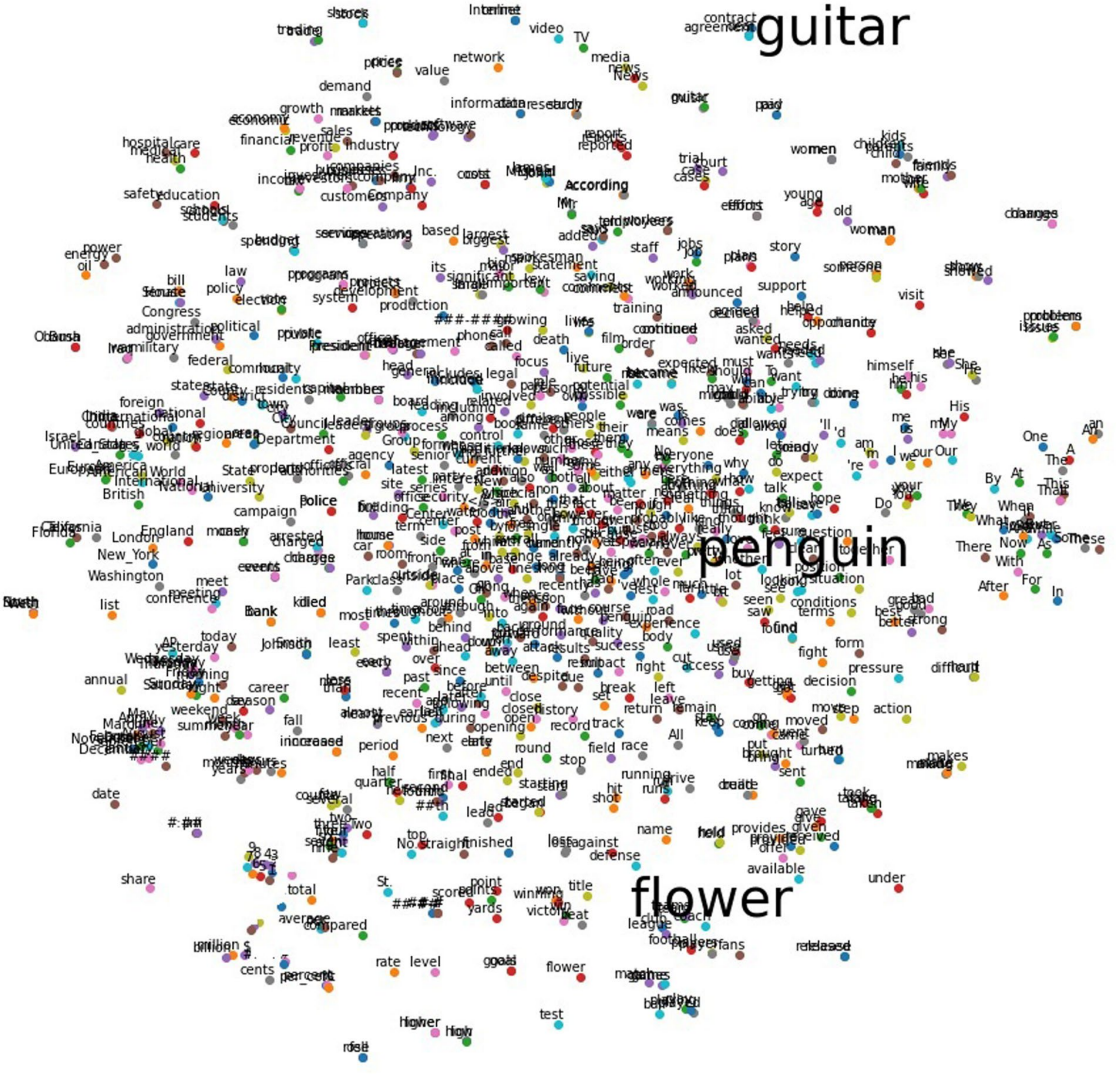
Visualisation demonstrating that the three selected semantic words (penguin, guitar and flower) are semantically distant from each other. The distances, computed using Word2Vec, are plotted in 2D using t-SNE.

This study involved six paradigm variations, consisting of two tasks: imagination or perception, and three sensory modalities: visual pictorial, visual orthographic and auditory comprehension. The semantic categories used were flower, penguin and guitar. These three categories were selected based on semantic distance and syllable length. Semantic distance was determined by computing a Word2Vec latent space^35^, where each word is represented as a vector and the distance between vector pairs signifies the semantic similarity of two words. The distance between each of the pairs was calculated to ensure all pair-distances were < 0.2. A visual plot was then created using a t-distributed Stochastic Neighbour Embedding (t-SNE) which enables high dimensional data to be visualised in a 2D space (see Fig. 2). While common daily objects may be preferred as stimuli for BCI purposes, we selected more obscure objects which are unlikely to be used in the same contexts. This decision was driven by two main factors. First, using objects that people encounter on a daily basis can introduce unpredictable semantic associations and relations from their daily routines. Secondly, objects we have expertise in processing, such as faces, may result in spatially clustered selectivity or brain modularity^36^. This can restrict the generalisability of findings to non-expertise categories and thereby reduce the overall scope of application. Another constraint in selecting the semantic categories was that they all have two syllables. It is crucial to keep syllable length constant in the auditory comprehension paradigm to ensure that decoding is based on semantic properties rather than the syllable number associated with different words.

#### Visual pictorial

**Fig. 3:**
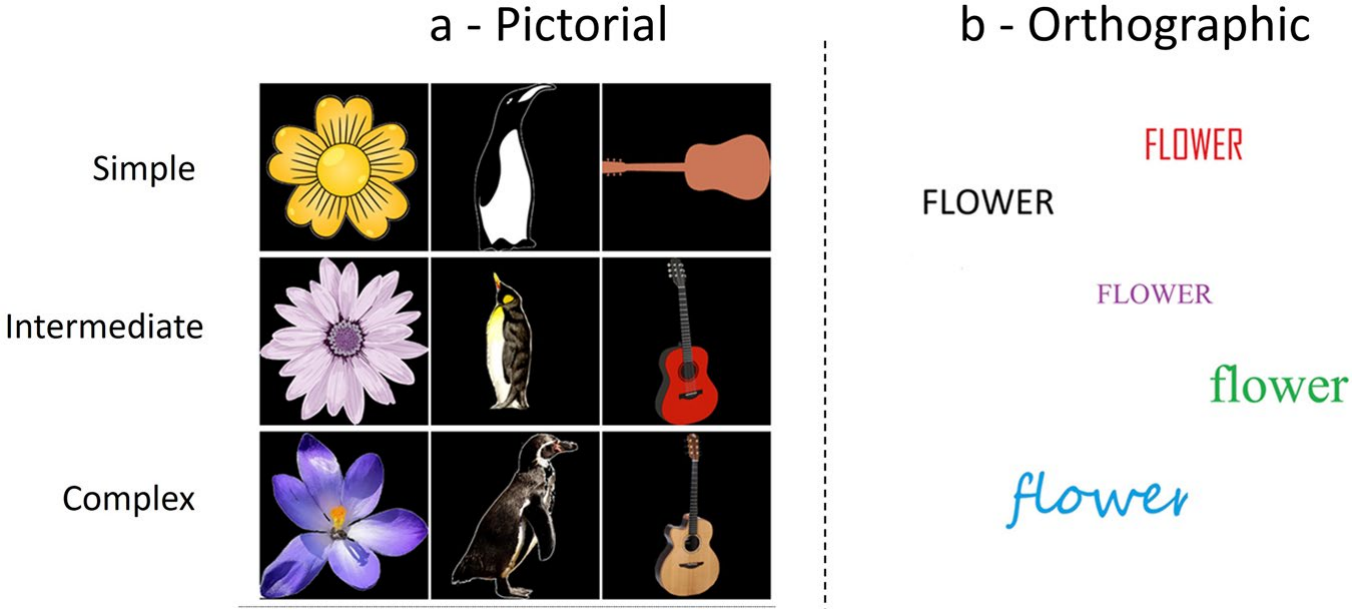
Examples of the visual (**a**) pictorial and (**b**) orthographic stimuli used in the experiment. Pictorial stimuli ranged in complexity from simple to intermediate to naturalistic, while orthographic stimuli varied in colour and font.

**Fig. 4:**
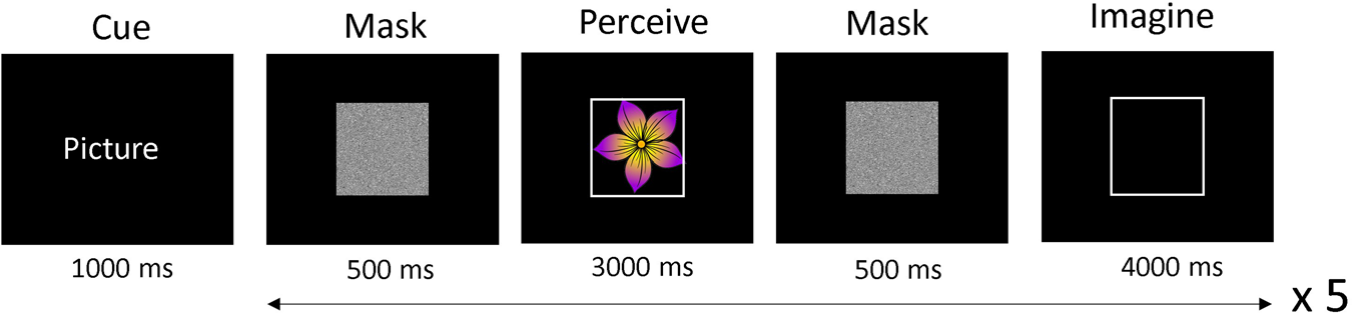
An example of a pictorial trial. After the cue, 5 trials occur with a different picture used in each. The picture is bounded in a white box, which reappears to frame the mental image for the imagination trial.

The visual pictorial paradigm involves perception and imagination of images belonging to the three semantic categories: flower, penguin and guitar. The visual pictorial stimuli consisted of coloured images with a resolution of 1200 × 1200 pixels against a black background (see Figs. 3, 4). In the context of object representation, incorporating objects within a consistent scene can enhance their semantic relations and aid in their recognition^37^. However, to maintain the purity of our study’s semantic concepts we opted to exclude any contextual scene information. This was because the addition of contextual information could potentially introduce unexpected semantic associations, thus introducing semantic noise. Furthermore, including contextual scenes would have added complexity, making the imagination task more challenging and potentially leading to increased participant fatigue. Therefore, we chose to focus solely on the objects themselves, without any accompanying contextual information. There are three levels of complexity for the images: simple, intermediate and naturalistic. For both flowers and guitars, there are eight different exemplars for the simple level and nine each of the intermediate and naturalistic levels. For penguin, there are nine exemplars for each level of complexity.

#### Visual orthographic

**Fig. 5:**
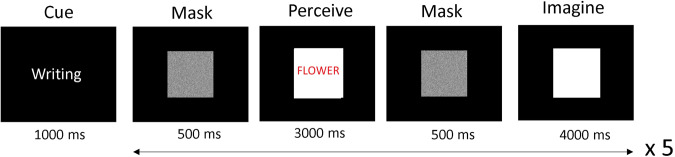
Example of an orthographic trial. After the cue, 5 trials occur with a different orthographic representation used in each. The written word appears against a white background, which reappears in the imagination trial to ensure similar scaling between imagination and perception.

Visual orthographic is the image of the word form of the semantic categories. The stimuli consisted of a 1200 × 1200 pixel white background with writing of either ‘penguin’, ‘flower’ or ‘guitar’ overlaid (see Figs. 3, 5). There were 30 exemplars for each category, with five different colours used (black, blue, red, green, purple) and six different font styles.

#### Auditory comprehension

**Fig. 6:**
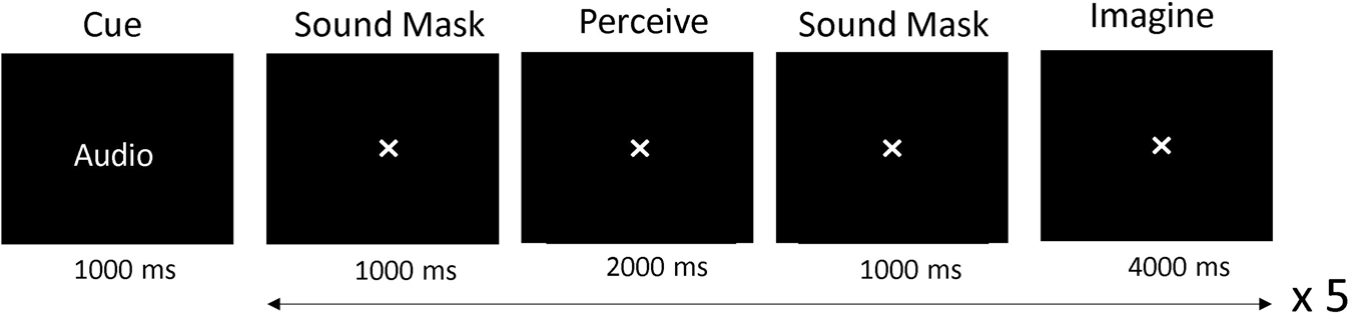
Example of an auditory trial. After the cue, 5 trials occur with a different spoken word recording used in each. A white noise sound mask of 1000 ms is used to prevent residual stimulus audio representation leaking between the perception and imagination trials.

Auditory comprehension consists of the speech version of the three semantic categories ‘penguin’, ‘guitar’ and ‘flower’. Recordings of these words were obtained from different speakers who did not participate in the EEG experiment. In the perception task, participants passively listened to these recordings which were processed using Audacity to remove background noise. Each clip was two seconds long. The words were spoken in either a normal, low or high voice. During the imagination task, participants were asked to imagine the spoken words that they had heard, using the same voice of the speaker rather than their own inner voice. To view an example of an audio trial, refer to Fig. 6.

### Data processing

#### Bad channels

To rigorously adjust for bad channels, a combination of manual and automatic bad channel detection was used. Bad channels identified from visual inspection of the plotted raw data in the ANT Neuro eego software were recorded in the *meta_extended.csv* file, discussed in the Data Records section, for each participant and session. Automatic bad channel detection was computed using PyPrep PrepPipeline https://pyprep.readthedocs.io/en/latest/generated/pyprep.PrepPipeline.html. This method utilises several bad channel detection methods, including identifying channels that do not correlate with other channels, channels with abnormally low or high amplitudes, or high quantities of high frequency noise, and channels with flat signals. Channels were re-referenced before interpolation was applied to correct for bad channels.

#### Re-referencing

During acquisition, electrodes were referenced to CPz. Re-referencing was conducted after all steps that offset the statistical trend of the overall data. Re-referencing was applied before and then after bad channel interpolation using common average referencing in MNE. A third re-referencing step was applied after filtering to remove low frequency drifts.

### Filtering

Data were filtered to remove power-line noise via notch filtering. Powerline noise in the UK where this dataset was recorded is at 50 Hz, therefore we filter for 50 Hz and its harmonics: 100 and 150 Hz. We also remove low frequency drifts which arise from movements of the head, scalp perspiration and wires. Filtering out frequencies below 2 Hz, via high pass filters, is recommended for high quality ICA decompositions^38^.

#### Artefact removal

Artefacts include eye movements such as blinks and horizontal eye movements as well as muscle activity. Independent Component Analysis (ICA) was applied to the raw pre-processed data rather than epoched data. The FastICA algorithm was used, and 50 components selected. To identify eye components, we used an MNE implementation to generate epochs around electrooculogram (EOG) artefact events. These were estimated from channels close to the eyes ‘Fp1’ and ‘Fp2’. By estimating these artefacts, the components can then be rejected from the ICA components. The resulting data after ICA retains all 124 original dimensions.

#### Epoching

Event labels for each condition were used to identify the beginning of each epoch. As the mne.Epochs() method to extract epochs from the raw data expects a consistent duration, we initially set tmin = 0 and tmax = 4. Subsequently, we use the known duration of each condition (see Table 2) to find the end points to properly epoch the data for the technical validation steps. We retain just the data relevant to perception and imagination, and keep only the additional data related to prior visual or auditory noise/mask, for the average event related potential analyses (see subsection Average Event Related Potentials).

**Table 2:** The duration in seconds for each type of epoch.

Task type	Sensory Modality	Duration
Perception	Auditory	2 s
Imagination	Auditory	4 s
Perception	Orthographic	3 s
Imagination	Orthographic	4 s
Perception	Pictorial	3 s
Imagination	Pictorial	4 s

## Data Records

**Fig. 7:**
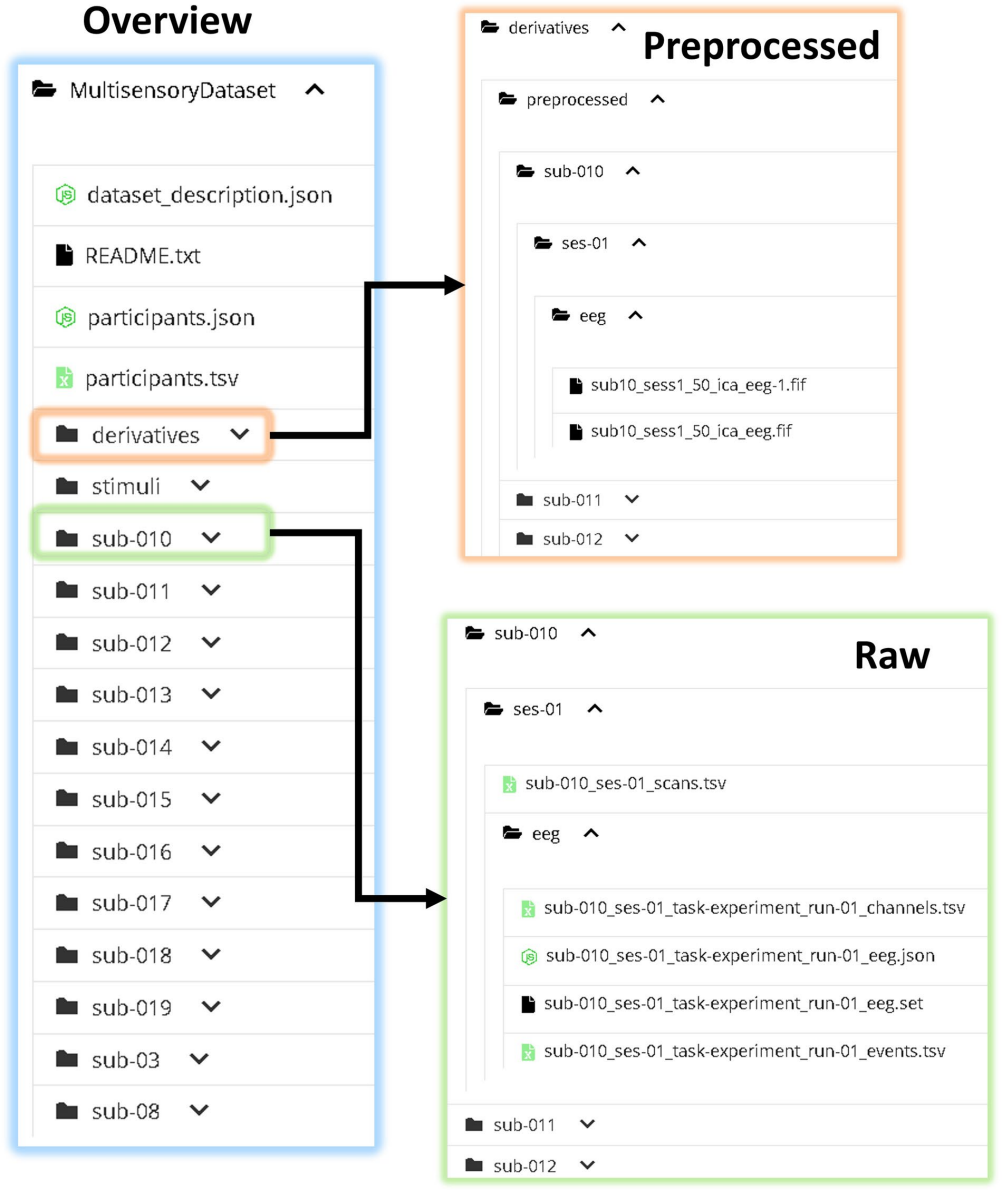
The directory structure of the data according to BIDS format. Two versions of the EEG data are provided, raw and pre-processed versions.

The full dataset^39^ can be accessed at the OpenNeuro repository (https://openneuro.org/datasets/ds004306/snapshot). The file structure and naming follows *Brain Imaging Data Structure* (BIDS) format (https://bids-specification.readthedocs.io/en/stable/). See Fig. 7. The participant with visual and hearing impairments is noted in the repository.

### Raw data

The original data produced in the ANT Neuro eego software are in *.cnt* and *.evt* format. They were converted in Matlab into *.set* and *.fdt* files to be in a format usable with the MNE package. A final conversion is computed to align the event data with BIDS format, resulting in a *.tsv* file. Therefore under the directories for each participant and session, i.e sub-01/ses-01/eeg/, are four files including the raw EEG data, the electrode data, the events data and a report file. The raw data are a continuous recording of one whole session. The event files have an event label for each specific stimulus used. The trial type provides information about the specific stimulus. For example, ‘Imagination_a_flower_high_5’ refers to the imagination audio condition in which a relatively high pitched voice saying the word flower is imagined and the specific voice id of this stimulus is ‘5’. An example of a visual event is ‘Perception_image_flower_c’ which refers to a perception of a flower picture. The ‘c’ indicates that the picture is relatively naturalistic/complex. Additionally, the start and end of the baseline obtained prior to the experiment tasks are provided.

### Preprocessed data

As seen in Fig. 7, the preprocessed data is formatted as *.fif* for each participant and session. Both the EEG data and the event data can be extracted from these files in MNE. The preprocessing pipeline that has been applied to the data is described in the Data Processing section.

## Technical Validation

### Average event related potentials

**Fig. 8:**
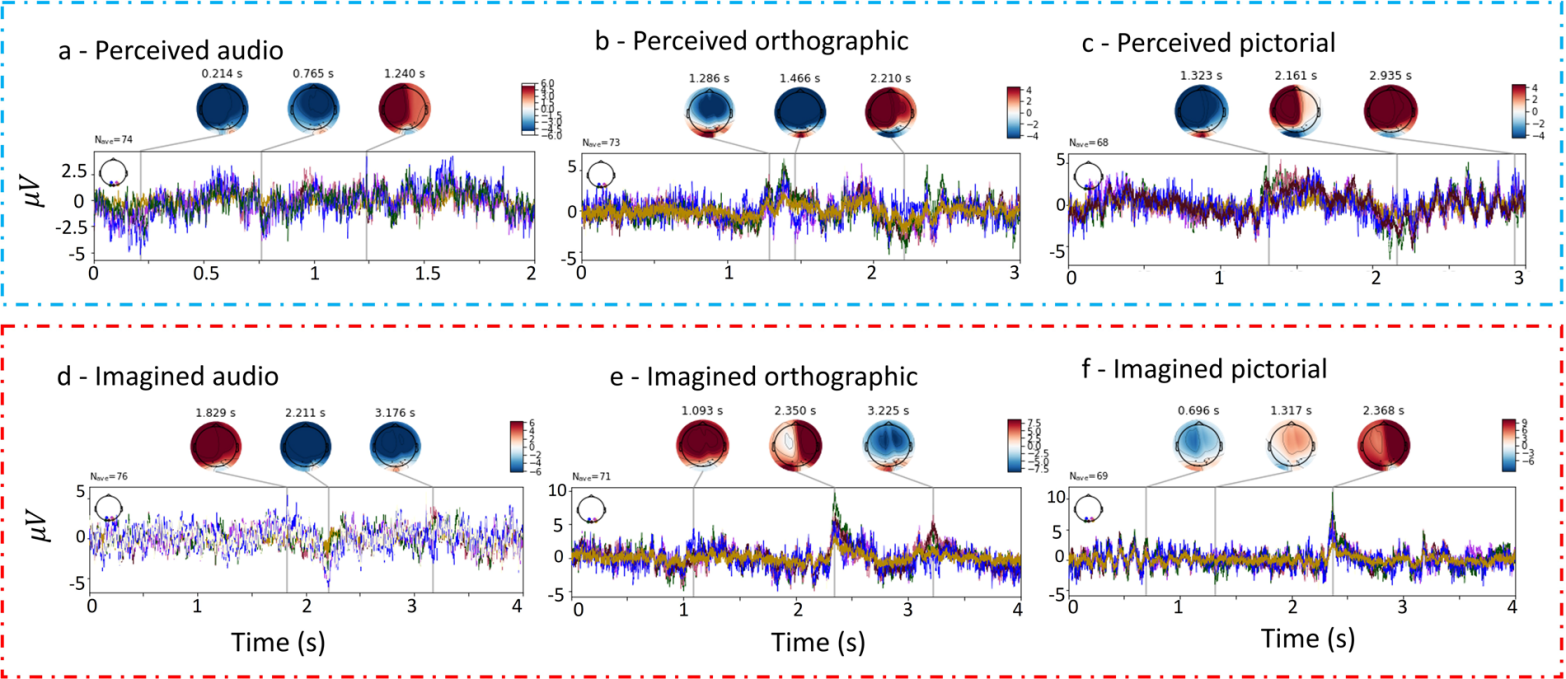
Displaying ERP for occipital regions including the electrodes: O1, O2, O1h, O2h, I1, Iz, I2, POO9, PO8, POO9b and POO10h. This is for participant 18, session 1.

Event Related Potential (ERP) plots can be used to investigate how the brain is modulated across time in response to specific stimuli. Averaging across trials shows consistent modulations. As there is high individual variance in neural anatomy and task strategy, we calculated the average ERPs for each participant and session separately. In Fig. 8, average ERPs for each of the six tasks for participant 18 from session 1 are shown. The selected electrodes for this analysis were in occipital and posterior regions. We can see that there is no consistent pattern for imagined audio. In contrast, there is a fairly consistent ERP across electrodes for the four visual conditions.

### Inter-trial coherence

**Fig. 9:**
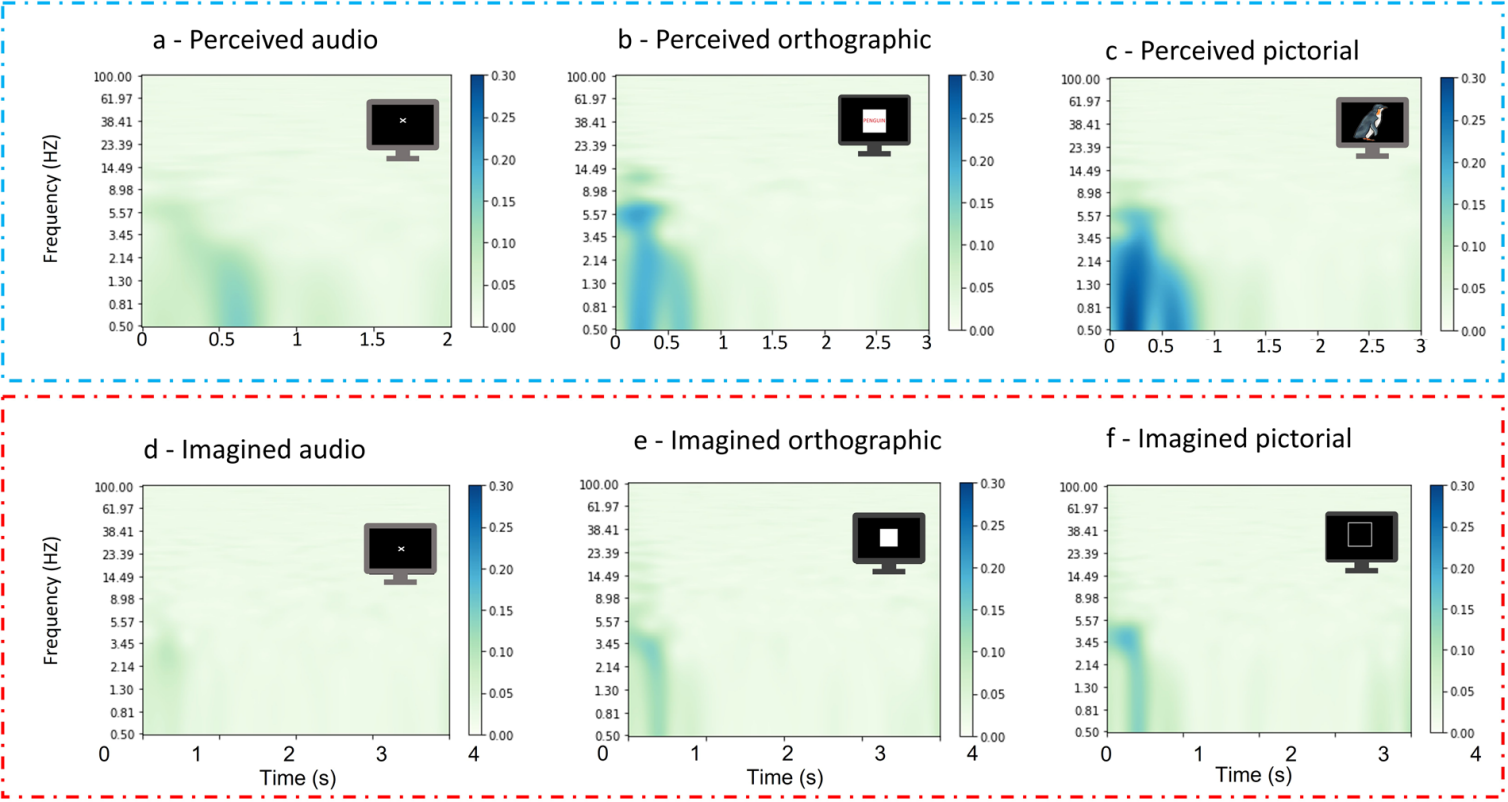
ITC for the six conditions averaged across participants. Specifically, ITC for (**a**) perceived audio, (**b**) perceived orthographic, (**c**) perceived pictorial, (**d**) imagined audio, (**e**) imagined orthographic and (**f**) imagined pictorial conditions. ITC is strongest in the perceived pictorial and orthographic conditions in the first 90 ms. ITC is weaker for imagination which is as expected due to the inter-trial variability in imagination generation and duration.

Inter-trial coherence (ITC) captures phase synchronisation or consistency across trials. A high ITC of 1 would indicate perfect coherence, whereas 0 is the lowest value and indicates no coherence. ITC is computed separately for each of the six conditions, and is shown here as the average across participants. In Fig. 9, it can be seen that there is stronger coherence for perception trials than for imagination trials. This is consistent with the expected increase of inter-trial and within participant variation in timing for generating imagined stimuli, whereas perceived stimuli have consistent onset and therefore higher ITC. Orthographic and pictorial perception both show strong ITC in the first 800 ms which likely relates to visual stimuli onset. Coherence is present but weaker in the same time window for imagined orthographic and pictorial tasks. Imagined audio has the least ITC, with a very weak ITC demonstrated in the first 500 ms.

### Averaged power spectral density

**Fig. 10:**
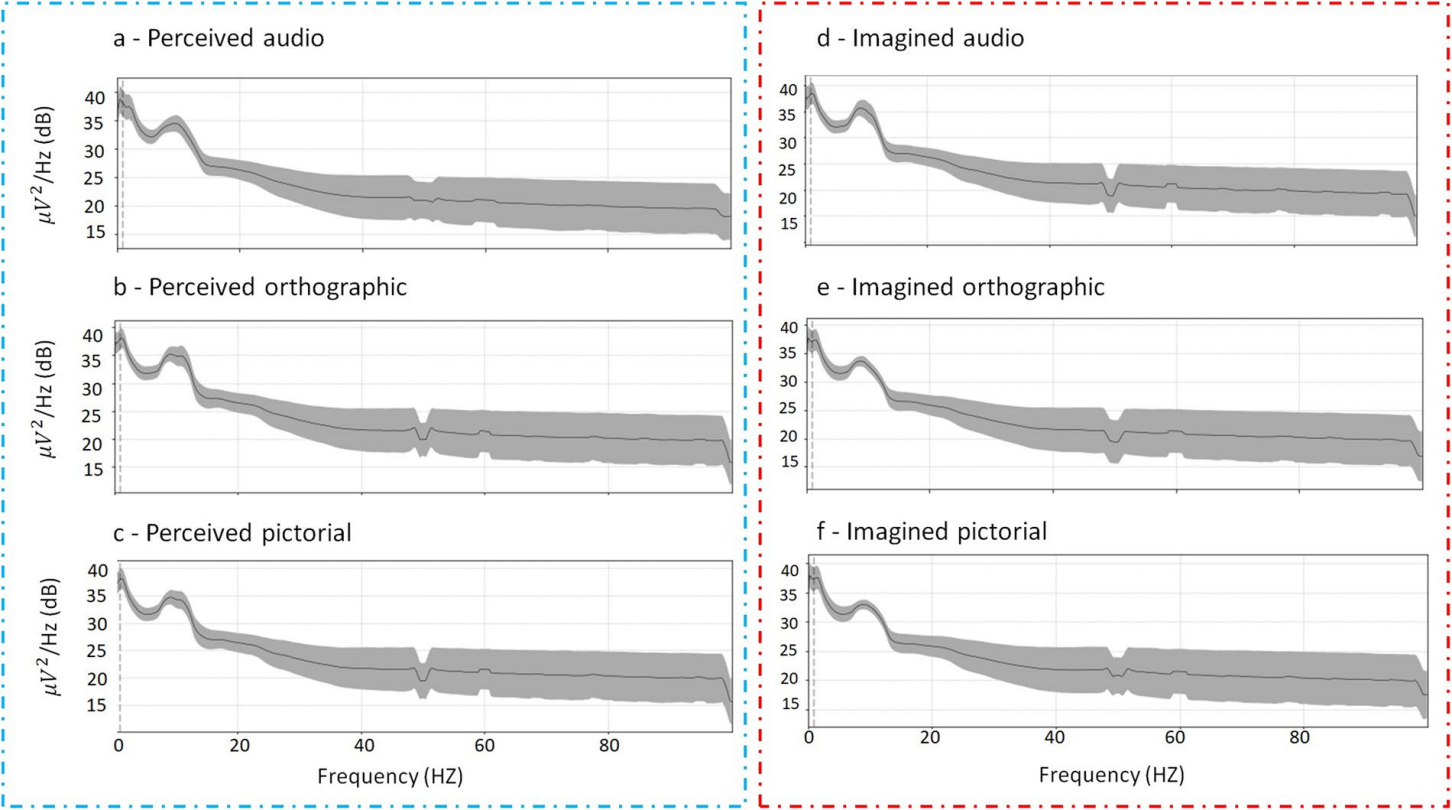
The average power spectral density averaged over the 124 trials and the participants for each of the six conditions (**a**) perceived audio, (**b**) perceived orthographic, (**c**) perceived pictorial, (**d**) imagined audio, (**e**) imagined orthographic, (**f**) imagined pictorial.

We report the power spectral density (PSD) averaged over participants to represent the distribution of signal frequency components (see Fig. 10). This is computed for each of the six tasks separately. In each task there is a strong alpha peak. The plot also demonstrates that the 50 Hz power-line noise has been successfully addressed via the notch filtering described in the Filtering section.

### Task classification

To demonstrate the feasibility of this dataset^39^ for decoding purposes and exploring neural mechanisms, we report a baseline performance on the imagination *vs* perception tasks for each sensory modality separately, using a logistic regression binary classification pipeline. For cognitive neuroscience, this gives insight into how distinct each task is for each modality. For BCI purposes, it can be useful to identify whether an individual is performing an imagination or perception task. To ensure consistency between the imagination and perception trials in terms of epoch length, we segmented all visual conditions into three second epochs and all the audio data into two seconds. In our analysis, we utilized a stratified cross-validation approach with five folds and conducted 50 iterations to ensure robustness of the results. The reported results (see Table 3) are averaged over the 50 iterations. Given that this was a binary classification task, chance level was set at 50%. For the visual modalities, classification accuracy is 75%. This is similar performance to that found in previous work^27^ in which 71% accuracy was achieved when classifying between whether a participant was imagining or observing pictures of flowers or hammers, using a SpecCSP classifier. In this current study, the average decoding performance of 60% accuracy between imagined audio and perceived audio is substantially lower. One potential explanation is that auditory perception and imagination have a higher degree of overlap than visual imagery and perception.

**Table 3:** Depicting classification accuracy between imagination and perception for stratified cross validation with five folds for each participant and session, averaged over 50 iterations.

Task and Classifier	3_3	8_3	10_1	11_1	12_1	12_2	13_1	14_1	14_2	15_1	15_2	16_1	17_1	18_1	19_1	Avg
Pictorial Imagination vs. Perception (LR)	0.66	0.7	0.78	0.71	0.73	0.71	0.83	0.67	0.73	0.92	0.87	0.78	0.93	0.63	0.84	0.77
Orthographic Imagination vs. Perception (LR)	0.6	0.66	0.8	0.76	0.78	0.73	0.86	0.66	0.72	0.81	0.82	0.84	0.94	0.64	0.83	0.76
Audio Imagination vs. Perception (LR)	0.48	0.59	0.59	0.57	0.62	0.53	0.63	0.59	0.55	0.55	0.63	0.63	0.72	0.55	0.71	0.6

LR refers to logistic regression. Here the participant number is before the underscore, and the session number after. For example, 3_3 is participant 3 and session 3.

**Table 4:** Depicts the number of trials for each overall task for each participant and session.

Task	3_3	8_3	10_1	11_1	12_1	12_2	13_1	14_1	14_2	15_1	15_2	16_1	17_1	18_1	19_1
Audio Perception	81	148	121	79	118	116	145	140	147	87	141	129	149	74	100
Orthographic Perception	85	149	116	71	119	97	142	138	146	79	143	118	147	73	102
Pictorial Perception	98	150	124	79	109	88	144	141	141	100	139	119	145	68	103
Audio Imagination	85	150	114	75	117	111	146	142	149	84	133	122	150	76	104
Orthographic Imagination	76	150	118	64	118	98	147	140	148	78	137	118	144	69	103
Pictorial Imagination	95	149	131	83	111	88	148	142	144	85	141	113	149	71	105

There is large variation in the number of trials completed out of 150, with a minimum of 64 and some participants completing all 150 trials. Here the participant number is before the underscore, and the session number after. For example, 3_3 is particpant 3 and session 3.

### Limitations and final remarks

We present a novel high resolution EEG dataset^39^ consisting of 124 channels. To the best of our knowledge, this is the first open source EEG dataset which captures not only semantic representation for several sensory modalities but also for both imagination and perception tasks for the same participant sample. This dataset is a promising starting point for investigating the feasibility of using semantic level representation for BCI input as well as enabling insights in cognitive neuroscience into the overlap in neural representation for semantic concepts in imagination, perception and different modalities. Still, decoding semantic representations from EEG data is difficult. To drive a representation related to semantic meaning rather than low level sensory details, we introduced high intra-class variance in this dataset. Intra-class variance results in more noise being present alongside the noise inherent from using EEG. Consequently, this is a challenging dataset for decoding, which makes it an interesting opportunity to apply deep learning techniques to extract meaningful information from noise. It is impossible to determine to what extent our participants were engaged with the experimental tasks, particularly for the imagination tasks. We included vividness metrics to indicate at minimum an individual’s capacity for imagery tasks. While this metric may be relevant for the decodability of sensory information, it is less likely to correlate with semantic representation. We anticipate that decoding accuracy will vary significantly between people. We hope that this dataset will create opportunities for other researchers to explore semantic decoding for BCIs as well as research questions related to neural mechanisms. By providing access not only to the raw data but also to the processed data and code for decoding, we offer a resource that can accelerate and support future research in these areas.

## Usage Notes

To facilitate the reproducibility and replicability of the study, the experiment was presented in Psychopy v.2021.2.3, a freely available software package. This ensures there are minimal barriers such as licences to prevent other researchers from using or modifying this experimental paradigm for their own studies. All code for processing and technical validation has been provided in a Jupyter Notebook tutorial style format so that following the steps for replication is as clear as possible, while also making it convenient for users to modify the code for related research questions. For example, minimal additional code is required to create classification pipelines for decoding semantics, tasks and modalities. File paths will need to be changed directly in the notebooks.

## Data Availability

The Psychopy files to compile the experiment are stored on the Github repository https://github.com/hWils/Semantics-EEG-Perception-and-Imagination. Also on this repository are the Python processing and technical validation scripts. Users can directly use the Python code provided 1) to compute preprocessing as described in this paper, and 2) to reproduce the experimental results presented in the technical validation section.
